# Integration of GWAS and transcriptome analysis to identify temperature-dependent genes involved in germination of rapeseed (*Brassica napus* L.)

**DOI:** 10.3389/fpls.2025.1551317

**Published:** 2025-03-03

**Authors:** Ruisen Wang, Guangyu Wu, Jingyi Zhang, Weizhen Hu, Xiangtan Yao, Lixi Jiang, Yang Zhu

**Affiliations:** ^1^ Institute of Economic Crop Sciences, Jiaxing Academy of Agricultural Sciences, Jiaxing, China; ^2^ Institute of Crop Science, Zhejiang University, Hangzhou, China; ^3^ Agricultural Experiment Station, Zhejiang University, Hangzhou, China

**Keywords:** *Brassica napus*, germination, temperature-dependent, cold tolerance, GWAS, transcriptome

## Abstract

Low temperature germination (LTG) is one of crucial agronomic traits for field-grown rapeseed in the Yangtze River Basin, where delayed sowing frequently exposes germination to cold stress. Because of its importance, the genetic basis underlying rapeseed germination under different temperatures has been continuously focused. By long-term field observation, we screened out two cultivars with significantly different LTG performance (JY1621 and JY1605) in field and lab conditions, which therefore were further used for the transcriptome sequencings at three key timepoints under normal and low temperatures. Comparative analysis among multiple groups of differentially expressed genes (DEGs) revealed a set of either early or late temperature response germination (ETRG or LTRG) genes, as well as cold-tolerant (CDT) and temperature-insensitive (TPI) candidate regulators at different germination stages. Furthermore, we performed a genome-wide association study (GWAS) using germination index of 273 rapeseed accessions and identified 24 significant loci associated with germination potential under normal temperatures. Through integrated analysis of transcriptome sequencing and GWAS, we identified a series of candidate genes involved in temperature-dependent germination. Based on the comprehensive analysis, we hypothesized that *BnaA3.CYP77A4* and *BnaA3.NAC078* could be important candidate genes for LTG due to their expression patterns and haplotype distributions. This study performed the multi-omics analysis on temperature-dependent germination and provided potential genetic loci and candidate genes required for robust germination, which could be further considered for low-temperature germination breeding of rapeseed.

## 1 Introduction

Rapeseed (*Brassica napus* L.) is one of the most important oil crops, which contributes over 13% of total vegetable oil production around the world (Tan et al., 2024). In China, the Yangtze River Basin, the world’s largest rapeseed-producing region, accounts for over 80% of the nation’s total rapeseed production (Tian et al., 2018, Tian et al., 2021). In recent years, within the Yangtze River cultivation regions, the extended rice growth period in the annual rice-rapeseed rotation system has delayed the sowing time of rapeseed, necessitating the use of cultivars with a shorter growth duration (Wang et al., 2022). And with the replacement of human labor by machine assistance, the traditional transplanting method has been progressively shifting to the direct sowing pattern (Zhu et al., 2021). Daily 15°C to 25°C range is considered as the optimal temperature for germination of rapeseed (Haj Sghaier et al., 2022). However, the delayed direct sowing can extend into late October or November, exposing germination in field conditions to low temperatures of around 10°C or even lower. Such low temperatures can severely impact germination and seedling development (Xian et al., 2017; Luo et al., 2021). The successful germination and subsequent seedling establishment are crucial for later vegetative and reproductive development. Therefore, identifying new genetic factors underlying temperature-dependent germination and seedling establishment is highly significant for ensuring the optimal growth of rapeseed.

Germination, as the beginning of the seed plants life cycle, is one of the most pivotal stages. It is commonly described as a complex process comprising three phases, including water absorption, metabolism reinitiation and the emergence of the radicle (Bewley, 1997; Finch-Savage and Leubner-Metzger, 2006; Rajjou et al., 2012). Under low-temperature stress such as less than 10°C, many cellular processes are disrupted at multiple levels, like membrane integrity, because cold temperatures reduce membrane fluidity, which would impair nutrient uptake and enzyme activity (Ding et al., 2019; Wu et al., 2024). The transformation from dry seeds to seedlings depends on both external factors including multiple environment factors and internal factors such as basic substance metabolism and plant hormone balancing (Weitbrecht et al., 2011; Han and Yang, 2015; Shu et al., 2015). Among various phytohormones, abscisic acid (ABA) and gibberellin (GA) are two major hormones that antagonistically regulate seed germination (Shu et al., 2016; Kim et al., 2022), and their content and transduction are sensitive to the ambient temperature (Shi et al., 2015). Low temperatures reduce the levels of GAs in plants by upregulating the expression of GA-inactivating genes, such as GA 2-oxidases (*GA2ox*), while simultaneously increasing ABA levels and activating the expression of ABA-responsive genes, including *ABSCISIC ACID RESPONSIVE ELEMENT-BINDING FACTOR 1* (*ABF1*), *ABF4/AREB2* and *ABA INSENSITIVE 5* (*ABI5*) (Lee et al., 2005; Achard et al., 2008). Additionally, auxin is also reported to play key roles in seed dormancy and germination (Wu et al., 2020; Li and Yu, 2022). In *Arabidopsis*, the YUCCA (YUC) family of flavin monooxygenases serves as key enzymes in the biosynthesis of auxin. And freshly harvested seeds of auxin-deficient *yuc1 yuc6* double mutants exhibit significantly decreased dormancy compared to wild-type seeds. *AUXIN RESPONSE FACTOR 10* (*ARF10*) and *ARF16* are auxin responsive genes, and single mutants of either *arf10* or *arf16* show reduced sensitivity to ABA during seed germination, which indicate that auxin regulates seed dormancy and germination synergistically with ABA (Liu et al., 2013). Despite progress in characterizing these pathways in different plant species, their regulation in allopolyploid crops like rapeseed with duplicated subgenomes and complex gene interactions remains poorly understood.

The multi-layer networks of low temperature response pathways in the model plant *Arabidopsis* have been intensively investigated (Ding et al., 2020). In *Arabidopsis*, approximately 4% to 10% of total coding genes annotated in the whole genome are regulated under various low temperature treatments, some of which play important roles and are further named as cold-responsive (*COR*) genes. Transcription factors (TFs) encoded by early *COR* genes regulate the expression of late *COR* genes, indicating a serious of transcriptional cascades (Lee et al., 2005; Park et al., 2015; Jia et al., 2016). When cold strikes, the expression of well-known AP2/ERF family TFs *C-REPEAT BINDING FACTORs/DEHYDRATION RESPONSIVE ELEMENT BINDING FACTOR 1s* (*CBFs/DREB1s*) are rapidly induced. And those TFs bind to C-repeat/DRE cis-elements in the promoters of downstream *CORs* to determinate the plant cold tolerance (Stockinger et al., 1997; Song et al., 2021). In recent years, with the development of sequencing technologies, multi-omics methods including RNA-sequencing (RNA-seq) and whole genome sequencing (WGS) are widely used to identify candidate genes. Low temperature germination (LTG) is a complex trait and is comprehensively regulated by multiple genetic loci (Wang et al., 2018). Many quantitative trait loci (QTLs) related to the LTG trait have been detected in many crops, such as rice, maize, and rapeseed (Wang et al., 2018; Luo et al., 2019; Zhang et al., 2020; Luo et al., 2021; Zhu et al., 2021). In rapeseed, bulked segregant analysis (BSA) is used to analyze the extreme accessions of LTG in the F2 population, identifying two major QTLs of LTG on chromosomes A9 and C1 (Zhu et al., 2021). Another study detects 22 significant loci associated with LTG through a genome-wide association study (GWAS) involving 442 rapeseed accessions (Luo et al., 2021). RNA-seq analysis between cold-tolerant and cold-sensitive rapeseed accessions detected various types of TFs, including *WRKY*, *bZIP*, *MYB*, *ERF*, and *NAC* families, which are associated with cold tolerance during germination and seedling establishment (Luo et al., 2019). However, current studies have yet to elucidate the molecular mechanisms underlying rapeseed germination across different temperatures and have not identified specific genes that contribute to LTG.

In this study, we identified two breeding cultivars with contrasting LTG performance (JY1621 and JY1605), and subsequent transcriptome analysis at three timepoints elucidated specific regulatory pathways involved in both normal-temperature and low-temperature germination. Meanwhile, GWAS based on the germination index of 273 natural accessions under normal-temperature revealed 337 candidate germination-related genes underlying 24 genetic loci. Subsequent comprehensive analyses identified several gene sets involved in regulating germination and seedling establishment under different temperature conditions. Combined with expression pattern analysis and haplotype investigation, we ultimately proposed that *BnaA3.CYP77A4* and *BnaA3.NAC078*, located within the peak on chromosome A3, would be promising candidate genes for enhancing LTG ability in rapeseed. Our results employed multi-omics approaches to dissect the regulatory pathways of rapeseed germination under different temperatures, and could provide valuable germplasm and potential genetic resources for improving LTG ability in rapeseed breeding in the Yangtze River Basin.

## 2 Materials and methods

### 2.1 Plant materials

Both of JY1621 and JY1605 are semi-winter cultivars from local breeding population. Seeds used for phenotypic observation and transcriptome experiment of JY1621 and JY1605 were harvested from strictly self-pollinating siliques in the field of Jiaxing Academy of Agricultural Science (Jiaxing, China). In previous studies, 991 rapeseed accessions from 39 countries around the world were collected and re-sequenced (Wu et al., 2019), and 300 accessions were selected to construct a core germplasm collection (Yan et al., 2020). In this study, 293 representative accessions as the core germplasm collection were used for seed germination experiments, and seeds of these accessions were harvested from Jiaxing Academy of Agricultural Science (Jiaxing, China) in 2023.

### 2.2 Seed oil content measurement and seed quality analysis

The measurement of seed oil content and analysis of seed quality traits of JY1621 and JY1605 were conducted using near-infrared spectroscopy (NIRS) methods described in the previous study (Hao et al., 2023) with some modifications. Briefly, mature dry seeds were harvested from strictly self-pollinating siliques. About three grams of seeds were placed in specific glass containers for scanning, and seed oil content measurement and seed quality analysis were conducted using the ANTARIS II FT-NIR Analyzer (Thermo Fisher Scientific, USA).

### 2.3 Germination treatment and sample collection of JY1621 and JY1605

One hundred intact seeds with uniform size of both JY1621 and JY1605 as a biological replicate were placed in a petri dish layered with two sheets of filter paper, and 10 ml of deionized water with daily supplementation of 1 ml was added to ensure consistent moisture content of the filter paper. Three biological replicates of each accession were used. To identify the germination difference between JY1621 and JY1605, prepared petri dishes of each cultivar were immediately moved to the indoor growth chamber with normal-temperature conditions (25°C 12 hour-light/20°C 12 hour-dark, 20,000 lux, 60% relative humidity), and another three petri dishes of each cultivar were transferred to a growth chamber with low-temperature conditions (10°C 12 hour-light/5°C 12 hour-dark, 20,000 lux, 60% relative humidity). Seed germination was defined as the emergence of a radicle exceeding 2 mm in length. Germination was monitored and recorded daily at Zeitgeber time 8 (ZT8). The germination of each cultivar was recorded for seven and fourteen consecutive days under normal and cold conditions, respectively.

Samples collection of JY1621 and JY1605 for transcriptome sequencing under 25°C and 8°C starts before imbibition. Three petri dishes of each cultivar were incubated in the growth chamber with 12 hour-light and 12 hour-dark conditions. After 8-hour (ZT8) and 36-hour (ZT12) treatment under 25°C and 8°C, another 24 samples were collected. Each sample was immediately frozen in liquid nitrogen and stored in -80°C freezer for total RNA extraction.

### 2.4 Transcriptome sequencing and data analysis

The 30 seed samples of JY1621 and JY1605 were sent to Biomarker Technologies Co., Ltd. (Beijing, China), where total RNA extraction, RNA quality examination, sequencing libraries construction, and transcriptome sequencing were done. After filtering out adaptor sequences and low-quality sequences, about 6-GB pair-end 150-bp cleaned reads were generated for each sample. Zhongshuang 11 (ZS11) is a semi-winter rapeseed cultivar mainly cultivated in the Yangtze River Basin, and its genome sequence has been wholly sequenced and assembled into a high-quality genome using PacBio SMRT long-reads sequencing technology (Song et al., 2020), which was named as ZS11.v0 and used as the reference genome in this study. The cleaned reads were aligned to ZS11.v0 using HISAT2 v2.2.1 (Kim et al., 2019). The raw read counts for each gene were calculated using featureCounts v2.0.6 (Liao et al., 2014) with pair-end counting mode. Pairwise differential expression analyses were performed using DESeq2 v1.42.0 (Love et al., 2014) with the input of raw read counts for each gene. Differentially expressed genes (DEGs) between two treatment groups were identified with the criteria adjusted *P*-value < 0.05 and | log_2_ (Fold change) | ≥ 1. After transferring the rapeseed genes into homologous genes of *Arabidopsis thaliana* using blast v2.15.0 (https://ftp.ncbi.nlm.nih.gov/blast/executables/blast+/LATEST/), gene ontology (GO) enrichment analysis of the DEGs was performed using R package clusterProfiler v4.10.0 (Wu et al., 2021). Fragments Per Kilobase of transcript per Million fragments mapped (FPKM) was used to normalize the abundance of gene expression. The principal component analysis (PCA) of all samples were performed using fast.prcomp module from R package gmodels v2.19.1 (https://cran.r-project.org/web/packages/gmodels/index.html) with the input of FPKM of each gene. The gene expression heatmaps were generated using the R package pheatmap v1.0.12 (https://cran.r-project.org/web/packages/pheatmap/index.html) with the input of FPKM of each gene. The visualization of PCA and DEGs was performed using R package ggplot2 v3.5.0 (https://ggplot2.tidyverse.org/).

### 2.5 Germination experiment and trait measurement of 293 rapeseed accessions

To compare the germination features of 293 rapeseed accessions, the germination experiment was performed under normal-temperature conditions as mentioned above. Three independent experiments were conducted for each accession, and 100 seeds with the uniform size were used in each experiment. Seed germination was recorded daily, and the germination rate recorded on the 7^th^ day was considered as the final germination rate for each accession. Subsequently, the germination index for each accession was calculated using the following formula described by the previous study (Liu et al., 2014).


$$\text{Germination index }(\text{GI})=\sum_{i=1}^{n}\frac{ɡi}{ti}$$

*gi* represents the number of seeds germinated on the i^th^ day, and *ti* represents the i^th^ day after sowing.

### 2.6 SNP calling and annotation

The raw resequencing data for rapeseed accessions from the core germplasm were obtained from the NCBI SRA database under the accession number SRP155312 (Wu et al., 2019). SNP calling was performed according to the previously described methods (Wu et al., 2019, Wu et al., 2024) with minor modifications. Briefly, all clean reads of 300 accessions from the core germplasm were mapped to the ZS11.v0 reference genome using MEM algorithm from BWA v0.7.17-r1188 (Li, 2014). The original bam files of all samples were sorted using SAMtools v1.19.2 (Li et al., 2009), and the duplicate reads of each bam file were marked using Picard module from GATK v4.5.0 (McKenna et al., 2010). Then, SNPs within 300 accessions were called using HaplotypeCaller module from GATK and were filtered using VariantFiltration module from GATK with the following parameters: QD < 2.0 || MQ < 40.0 || FS > 60.0 -clusterSize 2 -clusterWindowSize 5. The combined gVCF files and the final VCF file were generated using CombineGVCFs and GenotypeGVCFs module from GATK, respectively. After filtering out the variants that are not biallelic using VCFtools v0.1.16 (Danecek et al., 2011), a total of 13,698,593 SNPs were obtained within the 300 accessions by mapping reads to ZS11.v0 reference genome. SNP annotation was performed using ANNOVAR software (Wang et al., 2010) according to the genome sequence and gff3 file of ZS11.v0. Annotations of SNPs across the genome were categorized into six categories including exonic, intronic, intergenic, splicing site, 5’ or 3’-UTR and upstream or downstream of genes, and SNPs in exonic regions were further classified into synonymous SNPs or nonsynonymous SNPs.

### 2.7 Genome-wide association study of average germination index

In this study, 273 accessions with an average germination rate exceeding 80% were selected for the GWAS analysis. 2,540,225 high-quality biallelic SNPs were obtained by filtering out SNPs with minor allele frequency (MAF) less than 0.05 and genotype missing rate greater than 0.1 using the PLINK v1.90 (Purcell et al., 2007). To minimize the influence of regions with strong linkage disequilibrium, the SNP dataset was filtered with the parameter “–indep-pairwise 50 10 0.2” using PLINK v1.90, and the principal component analysis (PCA) was performed using the PCA module from PLINK v1.90. After the missing genotypes were imputed by BEAGLE v5.4 (Browning and Browning, 2009), GWAS was performed using GEMMA v0.98.1 (Zhou and Stephens, 2012) with a linear mixed model (LMM). The kinship matrix was calculated using PLINK v1.90, and the first two principal components of PCA were used as covariates for GWAS. The *P* value between each SNP and germination index was calculated, and -log_10_ (*P*) ≥ 5 was defined as the genome-wide threshold of significant loci. The genes located within 75kb upstream and downstream the significant loci were defined as candidate genes. The Q-Q plot and the Manhattan plot were generated using the R package CMplot v4.5.1 (Yin et al., 2021).

### 2.8 Linkage disequilibrium analysis and haplotype analysis

The Linkage Disequilibrium (LD) between SNPs in the 273 rapeseed accessions was evaluated using squared Pearson’s correlation coefficient (*r^2^*) in PLINK v1.90. The LDheatmap and regional Manhattan plot were generated using LDBlockShow v1.4.0 (Dong et al., 2021). The haplotype analysis of candidate genes was performed using CandiHap v1.3.2 (Li et al., 2023) on the basis of the average germination index and SNPs with annotation, and haplotypes containing homozygous alleles were used for phenotype classification.

### 2.9 Statistical analysis

Differences between the two groups were evaluated using a two-tailed Student’s *t*-test, with statistical analyses conducted in Excel 2021. For multiple group comparisons, one-way ANOVA followed by LSD multiple comparison tests were performed using IBM SPSS Statistics 27.

## 3 Results

### 3.1 JY1621 exhibited better low-temperature germination performance compared to JY1605

**Figure 1:**
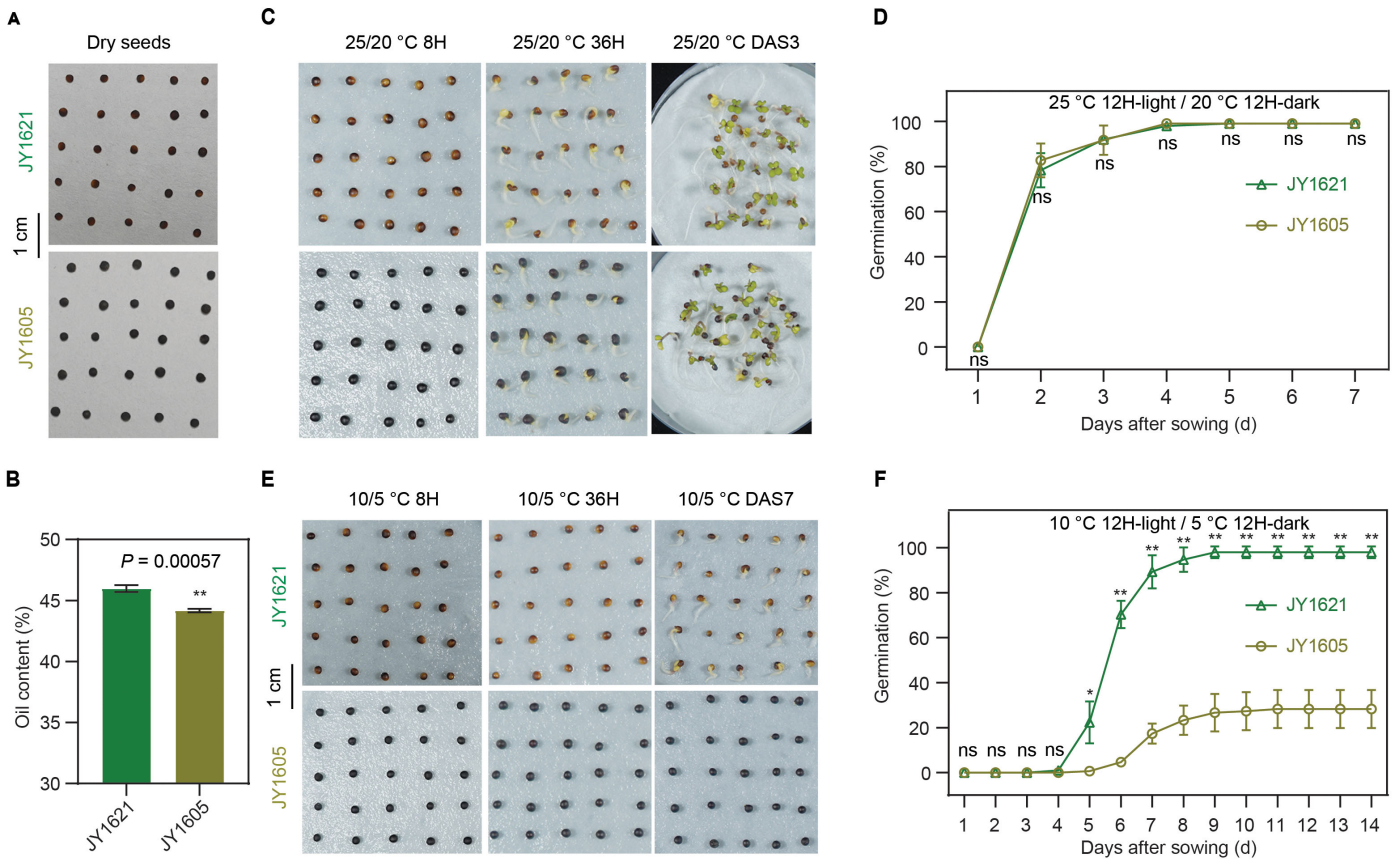
Germination comparison between semi-winter cultivars JY1621 and JY1605 under different temperatures. **(A)** Photos of dry seeds of JY1621 and JY1605. Seed coat color of JY1621 exhibited a mixture of yellow and brown while JY1605 had a pure black seed coat. **(B)** Seed oil contents of JY1621 and JY1605 measured by near-infrared spectroscopy (NIRS). **(C)** Photos of JY1621 and JY1605 germination at 8 hours (8H), 36 hours (36H) and 3 days after sowing (DAS3) grown under normal-temperature conditions. **(D)** Germination rates of JY1621 and JY1605 under normal-temperature conditions. **(E)** Photos of JY1621 and JY1605 germination after 8 hours (8H), 36 hours (36H), 7 days after sowing (DAS7) grown under low-temperature conditions. **(F)** Germination rates of JY1621 and JY1605 under low-temperature conditions. Scale bars represent 1 cm for **(A, C, E)**. Data are shown as means of three independent experiments ± SD In **(B, D, F)**. *P*-values are calculated by two-tailed Student’s *t* test: * indicates *P* < 0.05, ** indicates *P* < 0.01, and ns indicates no significant difference.

To study the genetic and molecular reasons underlying low temperature germination (LTG), we screened a genetic breeding population of semi-winter rapeseed in the JX field and selected out a cold-tolerant cultivar Jia You 1621 (JY1621) and another cold-sensitive cultivar Jia You 1605 (JY1605) during germination. For the seed phenotyping, the color of the JY1621 seed coat was yellow-brown mixture. By contrast, JY1605 was a normal, black-seeded cultivar with the dark black seed coat (**Figure 1A**). The seed oil content of JY1621 was significantly higher than that of JY1605 (**Figure 1B**; **Supplementary Table 1**), with few differences observed between these two cultivars in the yield-related or seed-quality traits (**Supplementary Figure 1**; **Supplementary Table 2**). To comprehensively investigate the differences in germination between JY1621 and JY1605, we evaluated the germination rates of two cultivars under normal- and low-temperature conditions in the controlled plant growth chamber. Under normal-temperature conditions (25°C 12-hour light/20°C 12-hour dark), there was no significant difference of germination rate between JY1621 and JY1605. And the final germination rates of both two cultivars were close to 100%, indicating that seeds harvested from their parents in the field had similar seed vigor (**Supplementary Table 3**). Furthermore, radicle protrusion and hypocotyl elongation were observed as expected at 36 hours after sowing (36H) in both JY1621 and JY1605. Subsequently, the hypocotyl and radicle continued to elongate, ultimately leading to the emergence of cotyledons inside out of the seed coat until 3 days after sowing (DAS3) (**Figures 1C, D**). Taken together, these data indicated that seeds of both two cultivars had high germination potential with low dormancy under normal conditions. However, under low-temperature conditions (10°C 12-hour light/5°C 12-hour dark), the growth rate was much lower, and the germination rates of JY1621 and JY1605 were quite different. On DAS7, the radicles of JY1621 had already protruded through the seed coats and well germinated, whereas the imbibed seeds of JY1605 had not yet exhibited radicle emergence (**Figure 1E**). Notably, the germination rate of JY1621 quickly got close to 100% in comparison only 20% of JY1605 germinated on DAS8 (**Figure 1F**; **Supplementary Table 4**). These findings demonstrated that under low temperatures, JY1621 exhibited significantly faster germination and higher seed germination rates than JY1605, indicating that JY1621 was a cold-tolerant cultivar at the germination stage.

### 3.2 Temperature-dependent and time-course transcriptome comparisons between JY1621 and JY1605

**Figure 2:**
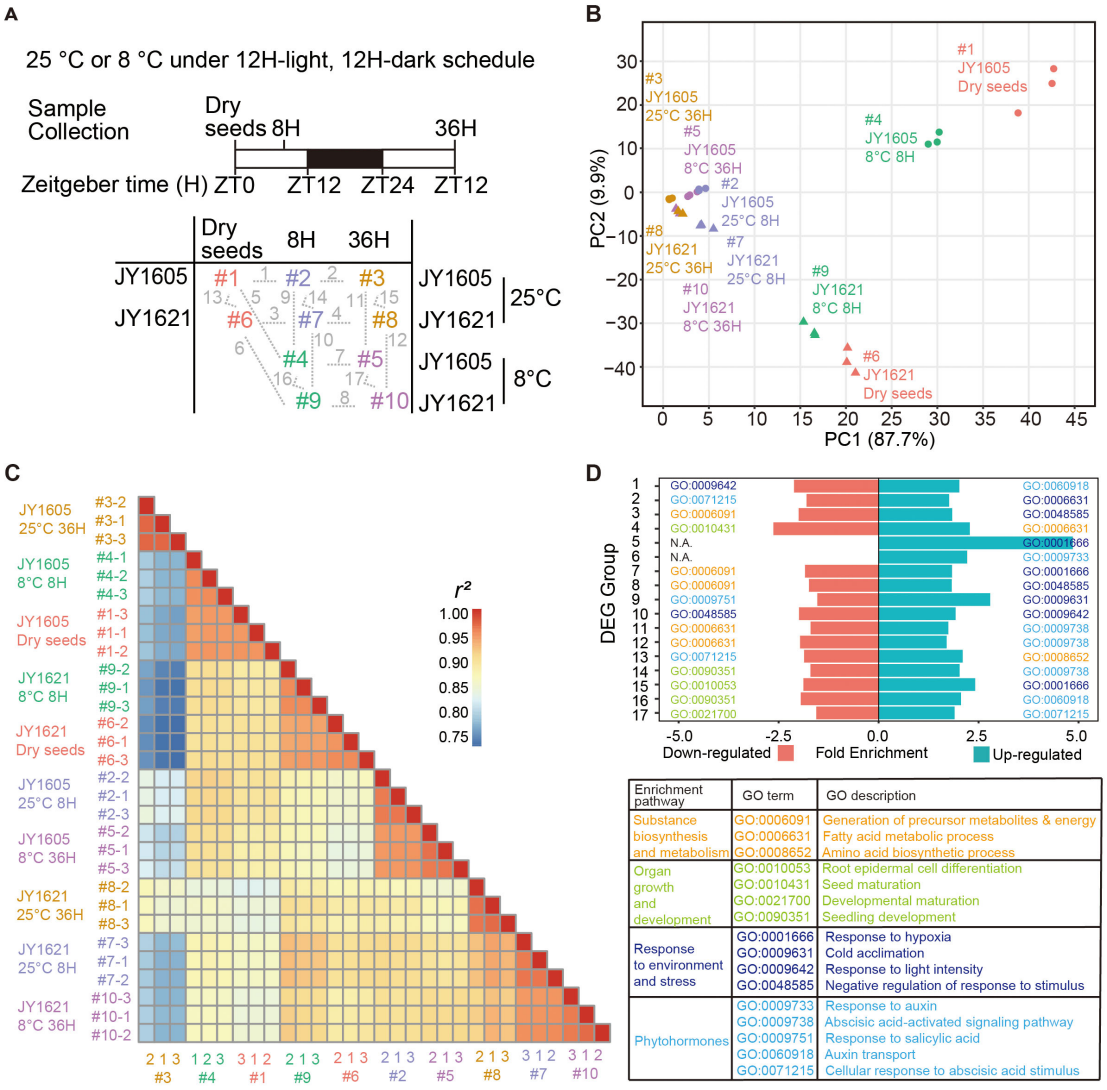
Transcriptome profiling of JY1621 and JY1605 germination under normal and cold conditions. **(A)** Schematic design of transcriptome sequencing experiments of JY1621 and JY1605 germination under different temperature treatments. Ten samples, labeled #1 to #10 in different colors, consisted of dry seeds (DS at ZT0), 8H imbibed seeds (8H at ZT8), and 36H imbibed seeds (36H at ZT12) collected at different growth stages under normal- and low- temperature conditions. For transcriptome analysis, seventeen one-to-one comparison groups were linked by grey dashed lines and further annotated from 1 to 17 in the bottom panel. ZT stands for Zeitgeber time. Samples of two cultivars under five conditions were annotated in pink, blue, orange, green and purple, respectively. **(B)** Principal component analysis (PCA) of 30 transcriptome samples. **(C)** Heatmap of Pearson correlation coefficient analysis with the input of Fragments Per Kilobase of transcript per Million fragments mapped (FPKM) of 30 samples. The color bar on the right represents the magnitude of correlation coefficient between each pair of compared samples. **(D)** Representative gene ontology (GO) terms and descriptions of 17 differentially expressed genes (DEGs) groups. N.A. represents no term was enriched in this group. Sample names are abbreviated in the figure. For example, 25°C 8H is short for the sample of imbibed seeds at 25°C after 8H growth on ZT8.

To further explore the underlying factors contributing to different germination performances between these two cultivars, we conducted a comprehensive transcriptome sequencing using JY1621 and JY1605 under normal- (25°C) and low- (8°C) temperature conditions. Dry seeds (ZT0), 8H imbibed seeds (8H on ZT8) and 36H imbibed seeds (36H on ZT12) of JY1621 and JY1605 grown under 25°C and 8°C conditions were collected in three biological replications (**Figure 2A**). After filtering out genes that were not expressed in all samples, the principal component analysis (PCA) and correlation analysis among 30 samples were performed by using Fragments Per Kilobase of transcript per Million (FPKM) of 86,120 genes (**Supplementary Tables 5**, **6**). The first principal component (PC1, 87.7%) and second principal component (PC2, 9.9%) could explain 97.6% of the total variances. In general, three biological replications were closely clustered and samples of two cultivars JY1621 and JY1605 were separated in the PC2 direction. From dry seeds to germinating seeds, samples were ultimately gathered in the PC1 direction. These results together demonstrated that transcriptome datasets contained differences from genomic specificities, developmental changes, and environment influences (**Figures 2B, C**). We conducted 17 comparative analyses of gene expression across the 10 datasets and identified differentially expressed genes (DEGs) for each group accordingly (**Supplementary Figure 2**; **Supplementary Tables 7**, **8**). Next, we performed gene ontology (GO) enrichment analysis by using the up-regulated genes and down-regulated genes from 17 comparison groups. Interestingly, the enriched GO terms could be divided into four major categories, namely substance biosynthesis and metabolism, organ growth and development, response to environment and stress, and phytohormones. Specifically, in the phytohormone category, processes associated with abscisic acids (ABA) including ABA signaling pathways (GO:0009738) and the cellular response to ABA (GO:0071215) were well known for their critical roles in germination. Additionally, processes related to auxin, such as response to auxin (GO:0009733) and auxin transport (GO:0060918), were also enriched in this category. In other categories, the enrichment terms like fatty acid metabolic process (GO:0006631), amino acid biosynthetic process (GO:0008652), seedling development (GO:0090351), and cold acclimation (GO:0009631) were particularly noteworthy (**Figure 2D**). These results suggested that genes involved in the processes of biosynthesis and metabolism of hormones and essential substances played a crucial role during seed germination under different temperature conditions.

### 3.3 Identification of genetic loci underlying normal-temperature germination variation by GWAS

**Figure 3:**
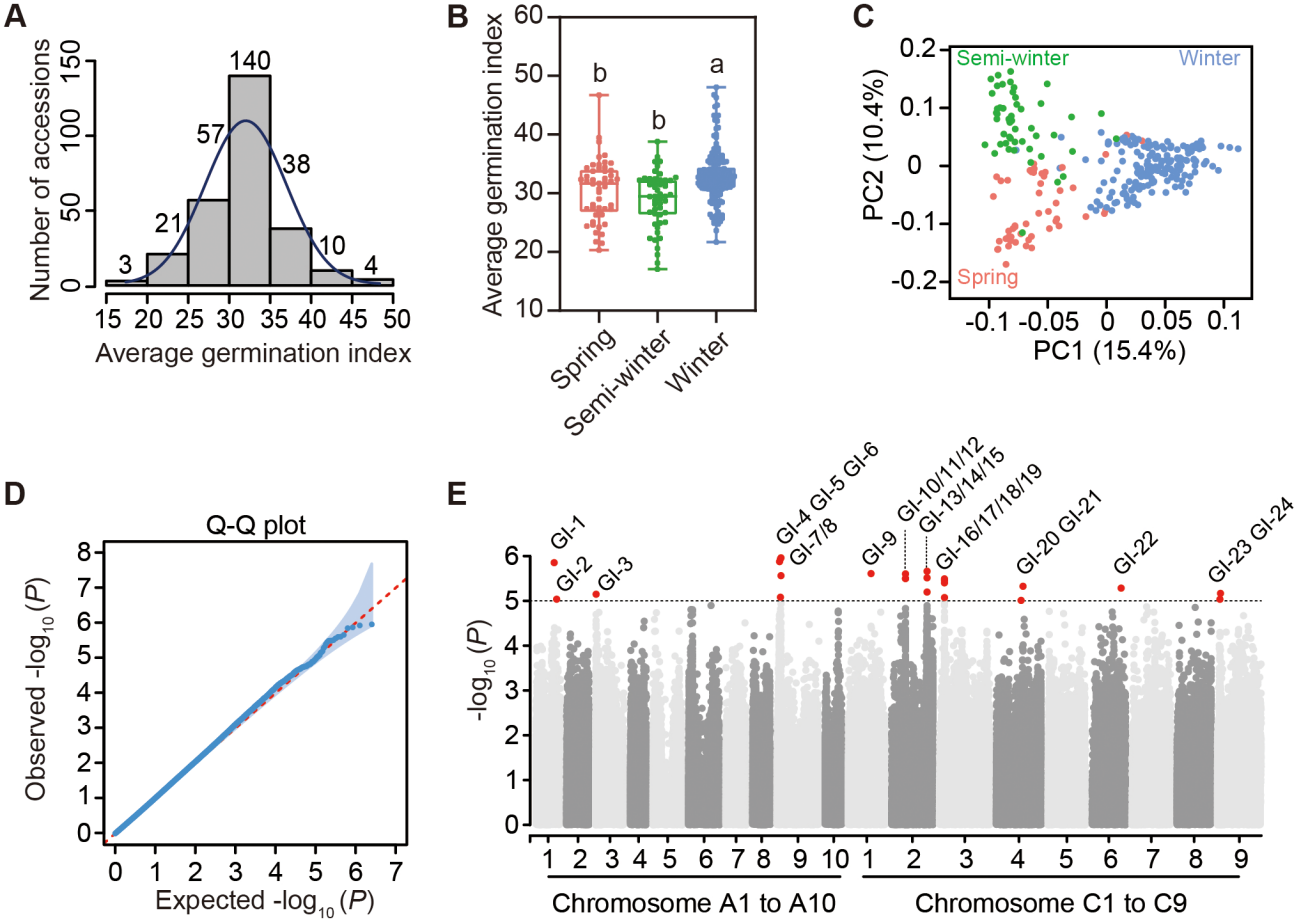
Genome-wide association study of germination variation among 273 rapeseed accessions. **(A)** Distribution of mean value of germination indices in three biological replicates among 273 accessions. **(B)** Comparison of average germination indices among three ecotypes. Different letters represent significant differences in the one-way ANOVA. **(C)** Principal component analysis (PCA) of SNP diversity among 273 rapeseed accessions. Spring, semi-winter and winter ecotypes are represented by pink, green and blue, respectively in **(B, C)**. **(D)** Q-Q plot of SNP deviation in genome-wide association study (GWAS) of average germination indices. The shaded areas in blue represent the deviation threshold of 0.05. **(E)** Manhattan plot of GWAS showing 24 highlighting QTLs in 19 chromosomes. *GI-1* to *GI-24* represent 24 SNPs with great significance exceeding the genome-wide control threshold (-log_10_ (*P*) ≥ 5).

In this study, we conducted the germination experiment of 293 rapeseed accessions from the core germplasm collection (Wu et al., 2019) under normal-temperature conditions (25°C 12-hour light/20°C 12-hour dark) to study the variations of germination among this natural population. To exclude the influence of seed vigor on germination, we used germination data of 273 accessions in this population for further analysis, as their average germination rates exceeded 80% (**Supplementary Tables 9**, **10**). The average germination index (GI) of three biological replicates of 273 accessions, calculated from DAS1 to DAS7, followed a normal distribution, ranging from a minimum of 17.08 in the R5131 accession to a maximum of 48.06 in the R4298 accession (**Figure 3A**; **Supplementary Table 11**). This population of 273 accessions contained 50 spring, 51 semi-winter and 172 winter ecotypes. The GI of winter ecotypes was significantly higher than that of spring and semi-winter ecotypes, while no significant difference was observed between the average germination indices of spring and semi-winter ecotypes (**Figure 3B**). After mapping the clean reads of each accession to the reference genome ZS11.v0 (Song et al., 2020), a total of 2,540,225 high-quality SNPs were obtained for subsequent analyses. Among all 19 chromosomes, the numbers of SNPs ranged from 70,353 on A10 to 234,030 on C3 (**Supplementary Figure 3**; **Supplementary Table 12**). The PCA result of SNP diversity among 273 accessions indicated that the principal component 1 (PC1) explained 15.4% of the total variances could distinguish the winter ecotype from both spring and semi-winter ecotypes and that PC2 explained 10.4% of the total variances could separate the semi-winter ecotype from the spring ecotype (**Figure 3C**). Based on high-quality SNPs among 273 accessions, we performed a genome-wide association study (GWAS) with the input of average GI and generated the Manhattan plot and Quantile-Quantile (Q-Q) plot for the visualization of germination associated genetic loci (**Figures 3D, E**). Finally, we identified 24 SNPs with great significance exceeding the genome-wide control threshold (-log_10_ (*P*) ≥ 5), namely *GI1* to *GI24*, as the significant genetic loci associated with germination in rapeseed. In details, these SNPs were widely distributed to 9 different chromosomes, namely A1, A3, A9 of the A subgenome and C1, C2, C3, C4, C6 and C9 of the C subgenome (**Figure 3E**; **Supplementary Table 13**). By examining a 75-kb interval upstream and downstream of the 24 significant SNPs, we screened out 337 candidate genes. The individual gene identification name and annotated information were listed in **Supplementary Table 14**.

### 3.4 Identification of early temperature responsive gemination genes

**Figure 4:**
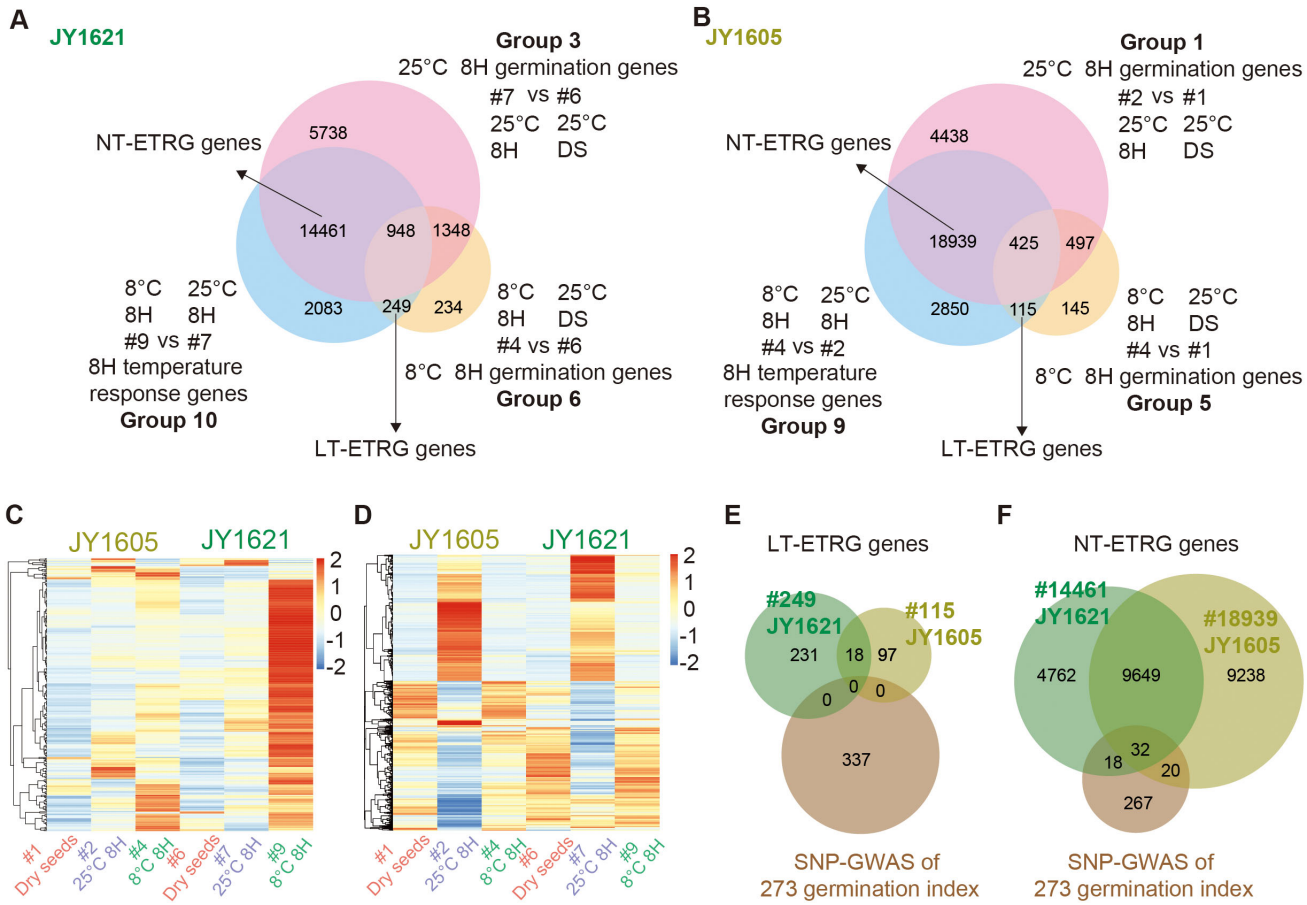
Identification and expression patterns of early temperature responsive gemination (ETRG) genes. **(A, B)** Venn diagrams of 8H response genes under different germination temperatures (Groups 10 and 9), 8H germination genes under 25°C (Groups 3 and 1) and 8H germination genes under 8°C (Groups 6 and 5) in JY1621 **(A)** and JY1605 **(B)** respectively, together indicate ETRG genes either under low-temperature (LT-ETRG, bottom) or under normal-temperature (NT-ETRG, left top) conditions. **(C, D)** Clustered heatmaps showing the normalized expression levels of totally 346 LT-ETRG genes **(C)** and 23,719 NT-ETRG genes **(D)** in the expression datasets of dry seeds (#1 and #6), 8H imbibed seeds under 25°C (#2 and #7) and 8°C (#4 and #9) of JY1621 and JY1605. **(E, F)** Venn diagrams representing the overlap between candidate genes from SNP-GWAS and LT-ETRG genes **(E)** and NT-ETRG genes **(F)** of JY1621 and JY1605.

To further explore the process of genes responding to different temperatures during germination stages, we conducted a comprehensive analysis of multiple comparison groups of transcriptome datasets. To exclude possible effects from dry seeds and identify early temperature responsive germination (ETRG) genes, three datasets of 8H germination genes under 25°C (Groups 3 and 1), 8H germination genes under 8°C (Groups 6 and 5) and 8H temperature response genes (Groups 10 and 9) were compared. Finally, 249 and 115 early response (8H response) genes under low temperatures (LT-ETRG), as well as 14,461 and 18,939 early response genes under normal temperatures (NT-ETRG) were identified in JY1621 and JY1605, respectively (**Figures 4A, B**; **Supplementary Tables 15**, **16**). To show the expression profiles of ETRG genes under different temperatures, the clustered heatmaps with the input of average FPKM of each gene were generated of 346 LT-ETRG and 23,719 NT-ETRG genes in both JY1621 and JY1605 (**Figures 4C, D**). Interestingly, most of genes had significantly higher expression levels in 8H imbibed seeds under 8°C of JY1621 than those of JY1605, including the well-known *COR* gene *BnaC8.CBF1 (BnaC08G0165900ZS)* and two homologous paralogs of *BnCBF2* (*BnaA03G0486800ZS*, *BnaA08G0172700ZS*), which was consistent with that JY1621 had a higher low-temperature germination rate than JY1605 (**Supplementary Table 15**).

To explore temperature-dependent germination genes in more details, we analyzed the intersection of candidate germination genes from the SNP-GWAS described above and ETRG genes identified in JY1621 and JY1605. Due to the small number of LT-ETRG genes, no overlapped gene was found between this dataset and candidate genes from GWAS (**Figure 4E**). By contrast, we successfully identified a total of 70 genes that overlapped between the SNP-GWAS candidate genes and the NT-ETRG genes. Among these, 32 genes were repeatedly shown in the NT-ETRG genes of JY1621 and JY1605, while 18 and 20 genes were specific to either JY1621- or JY1605-dataset of NT-ETRG genes, respectively (**Figure 4F**; **Supplementary Table 17**). Several WRKY-family TFs have been reported to be involved in seed dormancy and germination across various species (Wang et al., 2023). In the overlapping dataset, *BnaC9.WRKY30 (BnaC09G0059100ZS)* had relatively higher expression level in 8H imbibed seeds under 25°C of both JY1621 and JY1605. However, another WRKY transcription factor *BnaC9.WRKY18 (BnaC09G0072100ZS)* was highly expressed in 8H imbibed geminated seeds under 8°C of JY1605, while its expression level was lower in the corresponding sample of JY1621 (**Supplementary Figure 4**). These results suggested that different genes may perform distinct functions in the germination process depending on the temperature conditions.

### 3.5 Identification of late temperature responsive germination genes

**Figure 5:**
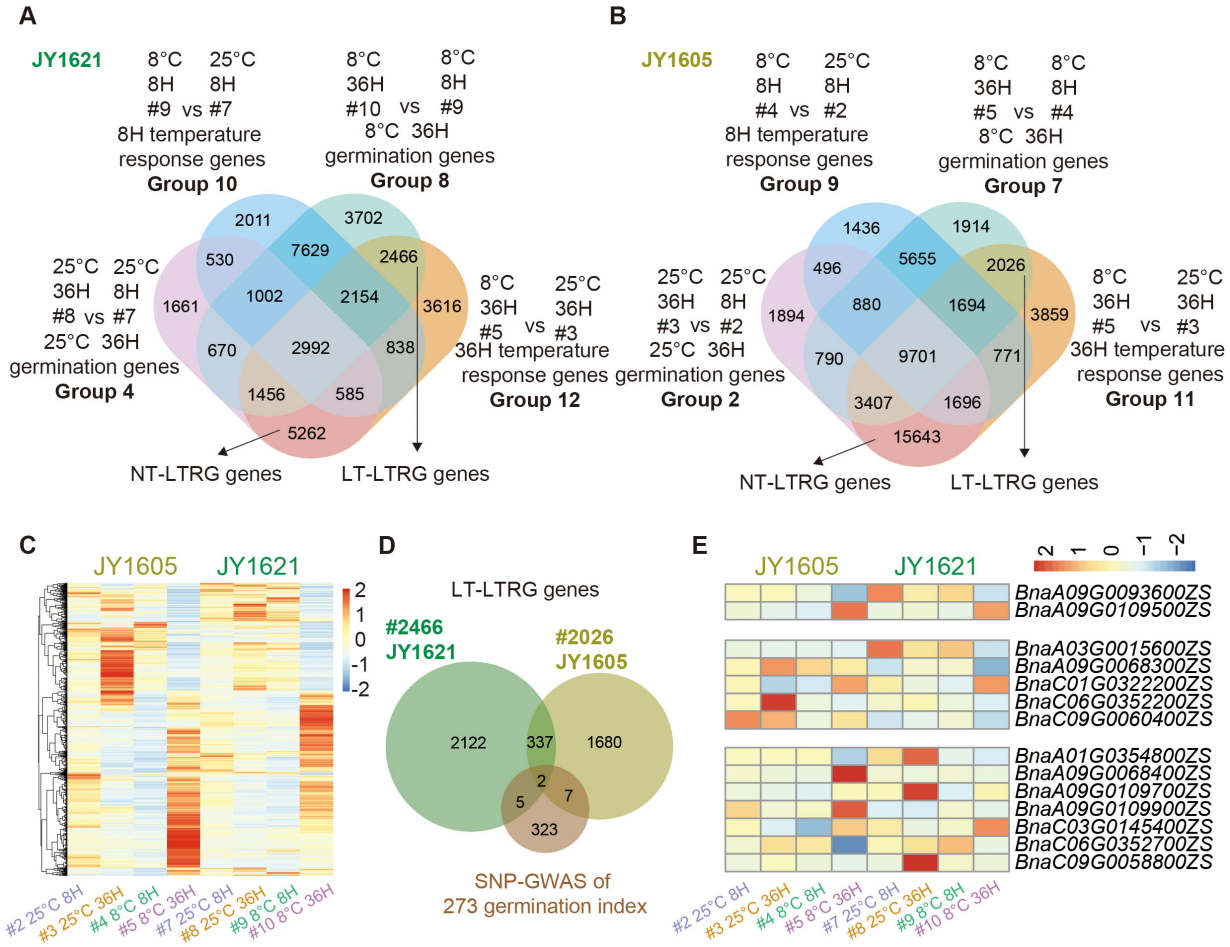
Identification and expression patterns of late temperature responsive germination (LTRG) genes. **(A, B)** Venn diagrams of 8H response genes under different germination temperatures (Groups 10 and 9), 36H response genes under different germination temperatures (Groups 12 and 11), 36H germination genes under 25°C (Groups 4 and 2) and 36H germination genes under 8°C (Groups 8 and 7) in JY1621 **(A)** and JY1605 **(B)** respectively, together indicate LTRG genes either under low-temperature (LT-LTRG, right top) or under normal temperature (NT-LTRG, bottom) conditions. **(C)** The clustered heatmap showing the normalized expression levels of totally 4,153 LT-LTRG genes in the datasets of imbibed seeds under 8H 25°C (#2 and #7), 8H 8°C (#4 and #9), 36H 25°C (#3 and #8) and 36H 8°C (#5 and #10) of JY1621 and JY1605. **(D)** The Venn diagram represents the overlap between candidate genes from SNP-GWAS and LT-LTRG genes of JY1621 and JY1605. **(E)** The heatmap showing the specific expression levels of 14 genes that overlapped between LT-LTRG genes and candidate germination genes from SNP-GWAS.

To explore the time-course response of germination-related genes to different temperatures, we compared the dataset of 36H temperature response genes (Groups 12 and 11) with the datasets of 36H 25°C response germination genes (Groups 4 and 2) and 36H 8°C response germination genes (Groups 8 and 7) in JY1621 and JY1605. After excluding the 8H temperature response genes, we identified 5,262 and 15,643 late response genes under normal temperatures (NT-LTRG) in JY1621 and JY1605, respectively. Additionally, 2,466 and 2,026 late response genes under low temperatures (LT-LTRG) were found in JY1621 and JY1605, respectively. (**Figures 5A, B**; **Supplementary Tables 18**, **19**). A total of 4,153 LT-LTRG genes were roughly divided into five clusters in the clustered heatmap. Notably, two homologous paralogs of late cold responsive gene *COR27* (*BnaA06G0444700ZS*, *BnaC04G0214400ZS*) were highly expressed in the 36H imbibed seeds under 8°C, especially in JY1621, indicating that prolonged exposure to low temperatures may lead to the accumulation of late cold-responsive gene expression (**Figure 5C**). It was interesting that a total of 17,788 NT-LTRG genes exhibited predominantly high expression levels in 36H imbibed seeds under 25°C, particularly in JY1605, demonstrating that the transition from germination to seedling establishment caused large changes in gene expression patterns (**Supplementary Figure 5A**).

To further identify the potential genes regulating temperature-dependent germination at the late germination stage, we similarly performed an integrated analysis of GWAS candidate genes and the datasets of LTRG genes (**Figure 5D**). We identified 14 shared genes between candidate genes from SNP-GWAS and LT-LTRG genes. In details, one bZIP transcription factor *BnaA9.bZIP23* (*BnaA09G0109900ZS*) was highly expressed in 36H imbibed seeds under 8°C of JY1605 while another bZIP transcription factor *BnaC6.TGA8* (*BnaC06G0352200ZS*) exhibited a lower expression level in 36H imbibed seeds under 8°C of JY1621 compared to other samples (**Figure 5E**; **Supplementary Table 20**). Additionally, 75 overlapping genes were identified between candidate genes from SNP-GWAS and NT-LTRG genes (**Supplementary Figure 5B**). Among these genes, one MYB transcription factor *BnaA9.MYB68* (*BnaC06G0352200ZS*) was highly expressed in 36H imbibed seeds under 25°C of JY1605 while the NAC transcription factor *BnaA3.NAC078* (*BnaA03G0014800ZS*) exhibited a very low expression level in 36H imbibed seeds under 25°C of JY1605. On the other hand, the auxin-induced gene *BnaA9.IAA9* (*BnaA09G0092300ZS*) showed a lower expression level in 36H imbibed seeds under 25°C of JY1621 compared to other samples (**Supplementary Figure 6**; **Supplementary Table 21**). These comprehensive analyses further identified candidate genes in JY1621 and JY1605 likely involved in temperature-dependent germination process at the late germination stage.

### 3.6 Identification of *Brassica napus* cold-tolerant and temperature-insensitive genes involved in germination

**Figure 6:**
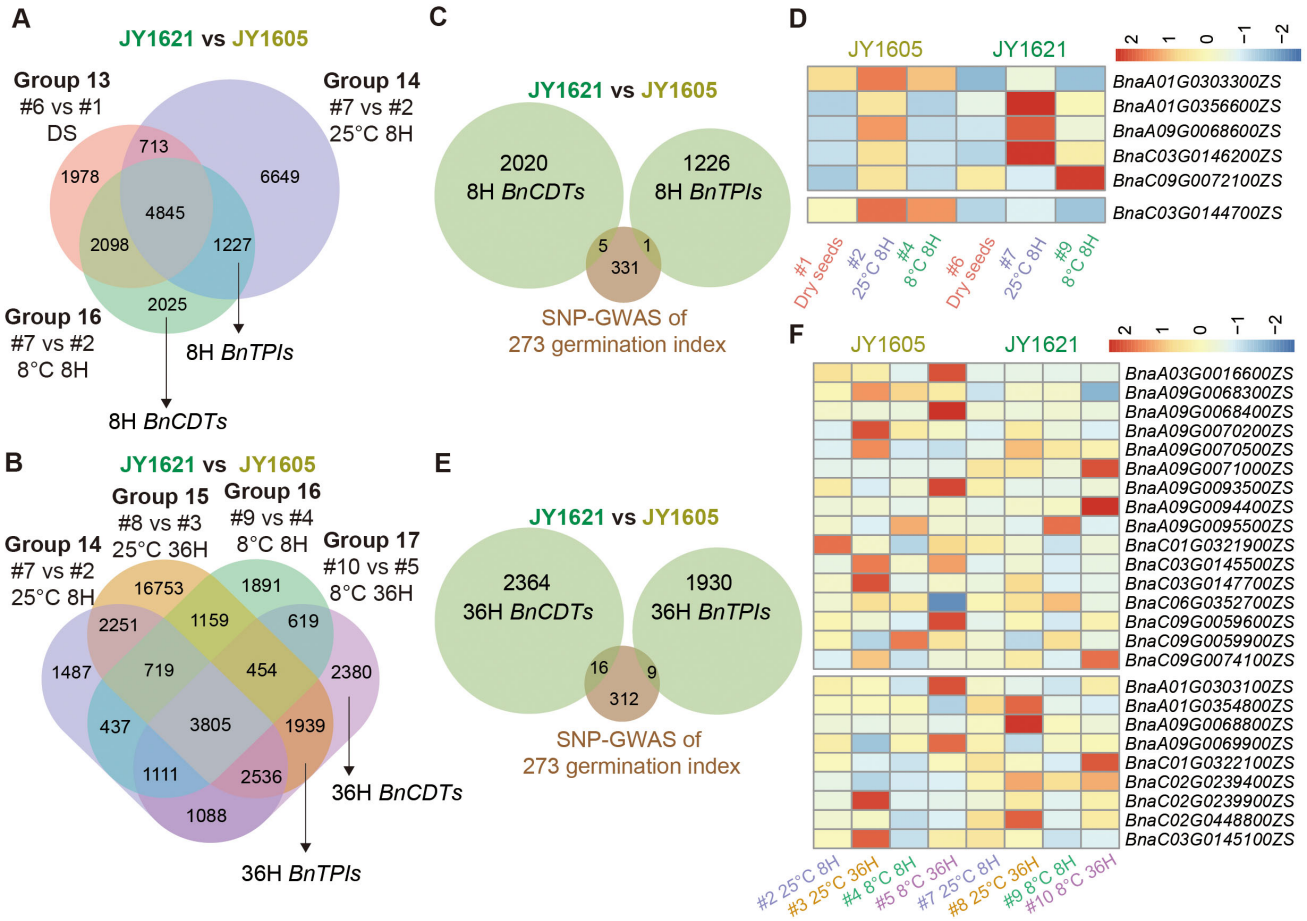
Identification and expression patterns of *Brassica napus cold-tolerant* (*BnCDT*) and *temperature-insensitive* (*BnTPI*) *genes* involved in germination. **(A, B)** Venn diagrams showing the identification of *BnCDTs* and *BnTPIs* at 8H **(A)** and 36H **(B)**, identified through five comparison groups of DEGs between JY1605 and JY1621. **(C)** Venn diagrams representing the overlapping genes among candidate germination genes of GWAS, *BnCDTs* and *BnTPIs* at 8H. **(D)** Expression patterns of 8H *BnCDTs* (top) and 8H *BnTPIs* (bottom) overlapping with candidate genes identified by GWAS. **(E)** Venn diagrams representing the overlapping genes among candidate germination genes of GWAS, *BnCDTs* and *BnTPIs* at 36H. **(F)** Expression patterns of 36H *BnCDTs* (top) and 36H *BnTPIs* (bottom) overlapping with candidate genes identified by GWAS.

After studying the time-course response genes under different temperatures during germination (ETRG and LTRG), we conducted five-condition comparisons (Dry seeds, 25°C 8 H, 25°C 36 H, 8°C 8 H and 8°C 36 H) and inter-group analyses by using ten datasets of gene expression levels between cold-tolerant JY1621 and cold-sensitive JY1605 cultivars to elucidate the reasons for their different germination performances under low-temperature conditions. Here we defined genes exclusively presented in the 8°C DEG groups as *Brassica napus* cold-tolerant *genes* (*BnCDTs*) during germination stage, whereas those genes appearing in both 8°C and 25°C DEG groups were defined as *Brassica napus temperature-insensitive genes* (*BnTPI*s). After excluding potential effects from other datasets, we identified 2,025 and 2,308 *BnCDT*s, as well as 1,227 and 1,939 *BnTPI*s in the 8H and 36H imbibed seeds, respectively (**Figures 6A, B**; **Supplementary Tables 22**, **23**). Next, we compared potential genes from SNP-GWAS with 8H *BnCDTs* and 8H *BnTPIs*, ultimately identifying five and one overlapping genes, respectively (**Figure 6C**; **Supplementary Table 24**). Among the 8H *BnCDTs* candidate genes, *BnaA1.GRF5* (*BnaA01G0356600ZS*) and *BnaC9.WRKY18* (*BnaC09G0072100ZS*) exhibited higher expression levels in 8H imbibed seeds at 8°C in JY1621 compared to JY1605, potentially contributing to the greater cold tolerance of JY1621. In contrast, the only overlapping 8H *BnTPI*, *BnaC3.FH5* (*BnaC03G0144700ZS*), which was involved in cytokinesis during endosperm development, showed higher expression in 8H imbibed seeds under both 8°C and 25°C in JY1605 compared to JY1621 (**Figure 6D**). We further compared candidate genes from GWAS with 36H *BnCDTs* and 36H *BnTPIs*, identifying 16 and 9 overlapping genes, respectively (**Figure 6E**; **Supplementary Table 25**). Among them, the 36H *BnCDT BnaC3.GH3.6* (*BnaC03G0145500ZS*) was an auxin-inducible gene and exhibited significantly higher expression in 36H imbibed seeds under 8°C in JY1621 compared to JY1605. Conversely, a VQ motif-containing protein *BnaA1.JAV1* (*BnaA01G0303100ZS*) was involved in the jasmonate-mediated defense response (Hu et al., 2013), and exhibited lower expression levels in 36H imbibed seeds under both 8°C and 25°C in JY1621 compared to JY1605 (**Figure 6F**). These results suggested that many genes involved in plant hormone response and regulatory pathways were likely critical regulators of temperature-dependent germination.

### 3.7 *BnaA3.CYP77A4* and *BnaA3.NAC078* were two candidate genes for temperature-dependent germination

**Figure 7:**
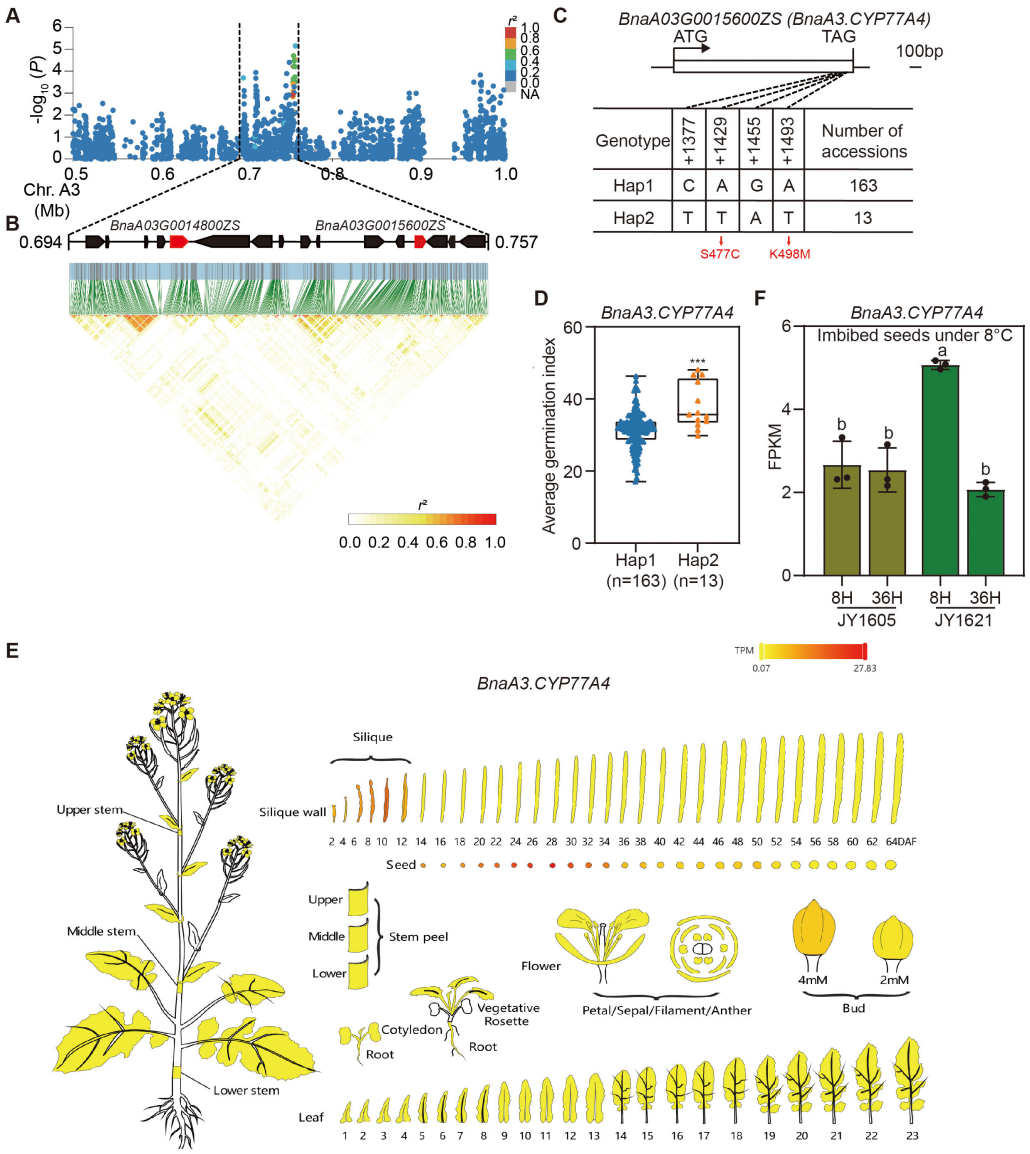
Haplotype analysis and expression patterns of *BnaA3.CYP77A4*. **(A, B)** Regional Manhattan plot **(A)** and LD heatmap **(B)** surrounding the peak located on chromosome A3 (*GI-3* in **Figure 3E**). Black dashed lines represent the candidate region for the peak. Solid red arrows represent two candidate genes. **(C)** Diagrammatic illustration showing the gene structure (top) and two main haplotypes (bottom) of *BnaA3.CYP77A4* using ZS11 as the reference. S477C and K498M represent two amino acid changes caused by nonsynonymous mutations. **(D)** Comparison of average germination indices under normal-temperature conditions between accessions with two different haplotypes of *BnaA3.CYP77A4*. The *P*-value is calculated by the two-tailed Student’s *t* test. *** indicates *P* < 0.001. **(E)** The tissue-specific expression pattern of *BnaA3.CYP77A4*. Data of expression levels in various tissues are obtained from BnIR (https://yanglab.hzau.edu.cn/BnIR/) (Yang et al., 2023). **(F)** Comparison of expression levels of *BnaA3.CYP77A4* in imbibed seeds under 8°C between JY1605 and JY1621. Different letters represent significant differences in the one-way ANOVA.

To accurately identify candidate genes associated with temperature-dependent germination in rapeseed, we conducted haplotype analyses of potential genes from SNP-GWAS. We found that the SNPs of candidate genes located on chromosome A3 were highly correlated with the germination index, so we mainly focused on the peak located on chromosome A3 (*GI-3* in **Figure 3E**). We investigated the 50 kb linkage region surrounding *GI-3* and identified *BnaA3.NAC078* (*BnaA03G0014800ZS*) and *BnaA3.CYP77A4* (*BnaA03G0015600ZS*) as the candidate genes for temperature-dependent germination (**Figures 7A, B**). In *Arabidopsis*, *AtCYP77A4*, a member of the cytochrome P450 family, encoded an epoxidase that catalyzed the epoxidation of various unsaturated fatty acids (FAs), including linoleic acid and α-linolenic acid (Sauveplane et al., 2009). Previous studies have found that *AtCYP77A4* regulates seed development and germination through auxin and lipid metabolism pathways in *Arabidopsis* (Kawade et al., 2018; Xiang et al., 2023).We screened for variations in the genomic sequences of *BnaA3.CYP77A4* and identified four linked SNPs in the coding region, two of which were nonsynonymous. The first nonsynonymous SNP at +1429 position changed the 477^th^ amino acid from serine to cystine (S477C), and the second at +1493 position changed the 498^th^ amino acid from lysine to methionine (K498M) (**Figure 7C**). Based on these four SNPs, allelic variations of *BnaA3.CYP77A4* among 273 rapeseed accessions were categorized into two main haplotypes. In the natural population, accessions with *BnaA3.CYP77A4* ^Hap1^ exhibited significant lower germination indices compared to those with *BnaA3.CYP77A4* ^Hap2^ under normal temperatures (**Figure 7D**; **Supplementary Table 26**). The expression data of various tissues from public database BnIR (Yang et al., 2023) indicated that *BnaA3.CYP77A4* was predominantly expressed in developing seeds, suggesting its potential role in regulating seed dormancy and germination in rapeseed (**Figure 7E**). Additionally, *BnaA3.CYP77A4* belonged to LT-LTRG genes identified above and exhibited higher expression levels in 8H imbibed seeds of JY1621 compared to JY1605 under 8°C (**Figure 7F**). These results demonstrated that *BnaA3.CYP77A4* may play a positive regulatory role in low-temperature germination. Another candidate gene *BnaA3.NAC078* was also categorized into two main haplotypes according to four linked SNPs in the genomic region, and accessions carrying *BnaA3.NAC078* ^Hap1^ showed lower germination indices than those with *BnaA3. NAC078* ^Hap2^ (**Supplementary Figures 8A, B**; **Supplementary Table 27**). In *Arabidopsis*, *AtNAC078* functioned as a transcriptional activator and regulated flavonoid biosynthesis under high-light conditions (Morishita et al., 2009). *BnaA3.NAC078* was widely expressed across various tissues, with particularly high expression levels observed in the mid to late stages of silique and developing seed (**Supplementary Figure 8C**). Furthermore, *BnaA3.NAC078* belonged to NT-LTRG genes identified above and exhibited significant higher expression levels in imbibed seeds of JY1621 compared to JY1605 at both 8H and 36H under 25°C (**Supplementary Figure 8D**), which indicated its role in regulating germination in normal temperatures. These results together demonstrated that *BnaA3.CYP77A4* and *BnaA3.NAC078* were two candidate genes for the SNP-GWAS and could potentially regulated germination of rapeseed under different temperatures.

## 4 Discussion

In this study, we identified two cultivars with different low-temperature germination (LTG) performance. By combining time-course transcriptome analysis with GWAS of germination potential under normal-temperatures, we identified multiple gene sets regulating germination under different temperature conditions. Further analysis pinpointed *BnaA3.CYP77A4* and *BnaA3.NAC078* as potential candidate genes for improving LTG. These findings fill the gap in understanding the temperature-dependent germination regulation mechanisms and may contribute to enhancing the LTG capacity of rapeseed in the Yangtze River Basin.

Current research on cold tolerance mechanisms during the germination and seedling stages of crops has largely focused on rice and maize, with relatively few studies conducted on rapeseed (Liu et al., 2018; Wang et al., 2018; Li et al., 2022). In this study, we clearly showed that a cold-tolerant cultivar JY1621 and a cold-sensitive cultivar JY1605 performed very differently at germination stage under low temperatures. One previous study conducted transcriptome sequencing between varieties with slow and fast germination speed under low-temperature conditions and identified 1,551 hub genes involved in low temperature germination (LTG). These DEGs include transcription factors from the WRKY, ERF, MYB, NAC and bZIP families, with significant enrichment in lipid metabolism pathways (Luo et al., 2019). In the present study, we performed the transcriptome sequencing on seeds of JY1621 and JY1605 at three timepoints under both normal- and low-temperature conditions. Further pairwise differential expression analyses identified 17 groups of DEGs, which were primarily enriched in fatty acid metabolism process and phytohormone response pathways, revealing that temperature variations during germination stage influence the metabolism of essential substances and the response process to various hormones. Furthermore, we conducted a comprehensive analysis of multiple DEG groups and identified genes respond to normal-temperatures (NT) and low-temperatures (LT) during both early (8H) and late (36H) stages of germination. Additionally, comparative analysis between JY1621 and JY1605 identified genes specifically differentially expressed under low-temperature conditions (*BnCDTs*), as well as those exhibiting differential expression across both low- and normal-temperatures (*BnTPIs*). Compared to the single screening and classification methods used in previous studies, the comprehensive and innovative classification approaches used in this study enables subsequent researchers to more effectively pinpoint genes that match their research focus.

In this study, the core germplasm utilized for GWAS was selected from a genetic population comprising 991 rapeseed accessions collected worldwide and subsequently re-sequenced, which displayed extensive genetic diversity (Wu et al., 2019; Yan et al., 2020). This core germplasm collection has been successfully applied to the identification of genes associated with traits including flowering time, petal size, leaf wax coverage and erucic acid content in seeds (Long et al., 2023; Xu et al., 2023; Wang et al., 2024; Xu et al., 2024). In the present study, average germination index of three biological replicates exhibited significant variations among 273 rapeseed accessions, with the data showing a normal distribution, making it well-suited for GWAS to identify potential genes associated with germination. We conducted a SNP-GWAS and identified 24 genetic loci significantly associated with germination potential under the normal-temperature condition, along with 337 candidate genes located within 75-kb upstream and downstream of these loci (**Figure 3**). A previous study crossed a cold-tolerant cultivar with a cold-susceptible cultivar to construct a mapping population consisting of 574 F_2_ progenies and two major-effect QTLs for LTG were detected on chromosomes A9 and C1 through BSA-seq (Zhu et al., 2021). However, these two QTLs did not overlap with the significant loci identified in this study, suggesting that the mechanisms regulating germination may differ under varying temperature conditions. Another study performed GWAS on 442 diverse rapeseed accessions, identifying 22 QTLs and 62 candidate genes associated with seed vigor under low-temperature stress (Luo et al., 2021). By comparing the candidate genes from both studies, we found that *BnPEX14* was present in both analyses (**Supplementary Table 14**), indicating that it would play a role in the germination process under different temperature conditions. In *Arabidopsis*, *AtPEX14* encodes a peroxisomal biogenesis factor that plays a crucial role in the peroxisomal protein import machinery. Mutations in *AtPEX14* result in reduced enzyme levels across various peroxisomes, leading to defects in fatty acid degradation and abnormal peroxisomal morphology (Hayashi et al., 2000). These findings suggest that the production and metabolism of fatty acids and peroxisomes are crucial in temperature-dependent germination, which is consistent with the GO enrichment terms revealed in this study, specifically fatty acid metabolic process (GO: 0006631) and response to hypoxia (GO: 0001666).

By integrating GWAS with transcriptome analysis, we identified several candidate genes involved in temperature-dependent germination in rapeseed. For example, *BnaC9.WRKY18* was identified as one 8H *BnCDT* in this study and exhibited a significant higher expression level in 8H imbibed seeds of JY1621 compared to JY1605. Its ortholog in *Arabidopsis*, *AtWRKY18*, negatively regulated seed germination and root growth under ABA treatment (Chen et al., 2010), indicating that *BnaC9.WRKY18* may play a role in regulating the early stage of rapeseed germination under low-temperatures. *BnaC9.WRKY30* was identified as a candidate gene of *GI-23* and showed high expression levels in both 8H imbibed seeds under normal-temperatures and 36H imbibed seeds under low-temperatures. In *Arabidopsis*, overexpression of its ortholog *AtWRKY30* could enhance the tolerance to oxidative and salinity stresses during germination (Scarpeci et al., 2013), suggesting its regulatory role in rapeseed germination under varying temperature conditions. In addition, one *Calmodulin-regulated Receptor-Like Kinase 1* in C3 (*BnaC3.CRLK1*) was identified as one 36H *BnTPI* and a candidate gene underlying the peak on chromosome C3. In *Arabidopsis*, *AtCRLK1* was reported to play an important role in regulating cold tolerance at the seedling stage (Yang et al., 2010), implying that it may serve as a regulatory factor that integrated temperature responses and germination in rapeseed. Previous studies have shown that ABA and GA are two key phytohormones that antagonistically regulate seed germination under various conditions (Shu et al., 2016; Kim et al., 2022). In this study, we discovered that DEGs identified in the transcriptome were enriched in GO terms related to auxin including response to auxin (GO: 0009733) and auxin transport (GO: 0060918) (**Figure 2D**). Additionally, several genes involved in the auxin signaling pathway, such as *BnaA3.CYP77A4*, *BnaA9.IAA9* and *BnaC3.GH3.6* (Fujita et al., 2012; Casanova-Saez et al., 2022; Xiang et al., 2023) played various roles in rapeseed germination under different temperature conditions, demonstrating that auxin was also an essential hormone for the interaction between plants and their environment during germination. However, the detailed genetic functions and molecular mechanisms of these candidate genes involved in temperature-dependent germination require further investigation. Taken together, this study provided valuable genetic resources for temperature-dependent germination and could give the theoretical guidance for the breeding of cold-tolerant rapeseed cultivars.

## Data Availability

The datasets presented in this study can be found in online repositories. The names of the repository/repositories and accession number(s) can be found in the article/**Supplementary Material**.
