# Supplementary material for: Integration of GWAS and transcriptome analysis to identify temperature-dependent genes involved in germination of rapeseed (Brassica napus L.)
Source: Front Plant Sci. 2025 Mar 3;16:1551317. doi: 10.3389/fpls.2025.1551317 (PMC11911475; doi:10.3389/fpls.2025.1551317)
Supplement: Supplementary file 5 [file DataSheet5.docx]

**Supplementary File**

**Integration of GWAS and transcriptome analysis to identify temperature-dependent genes involved in germination of rapeseed (*Brassica napus* L.)**

**Ruisen Wang^1+^, Guangyu Wu^2+^, Jingyi Zhang^2^, Weizhen Hu^3^, Xiangtan Yao^1^*, Lixi Jiang^2^* and Yang Zhu^2^***

^1^ Jiaxing Academy of Agricultural Sciences, Jiaxing, China

^2^ Institute of Crop Science, Zhejiang University, Hangzhou, China

^3^ Agricultural Experiment Station, Zhejiang University, Hangzhou, China

**^+^**Equally contributed first authors

*Co-corresponding authors

*E-mails:

Xiangtan Yao, [yxt156@hotmail.com](mailto:yxt156@hotmail.com)

Lixi Jiang, [jianglx@zju.edu.cn](mailto:jianglx@zju.edu.cn)

Yang Zhu, [zhuyang2020@zju.edu.cn](mailto:zhuyang2020@zju.edu.cn)

**Supplementary Figures**

**
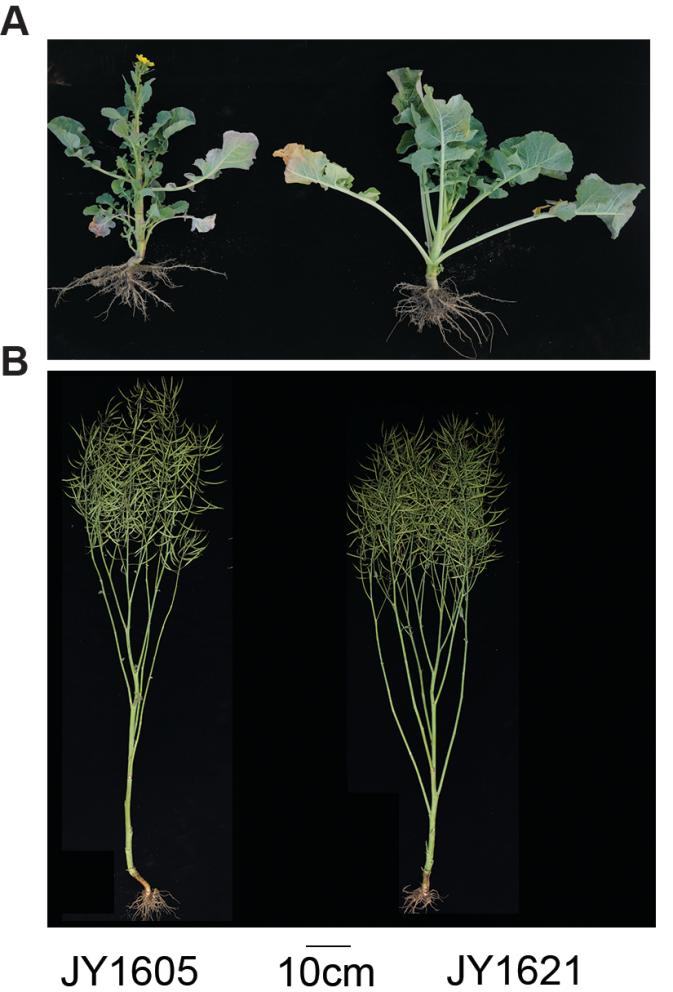
**

**Supplementary Figure 1. Plant photos of JY1621 and JY1605 in different growth stage.**

**(A, B)** photos of 15-weeks-old seedlings (**A**) and silique maturation stage (**B**) of JY1621 and JY1605 grown in field. The scale bar represents 10 cm.


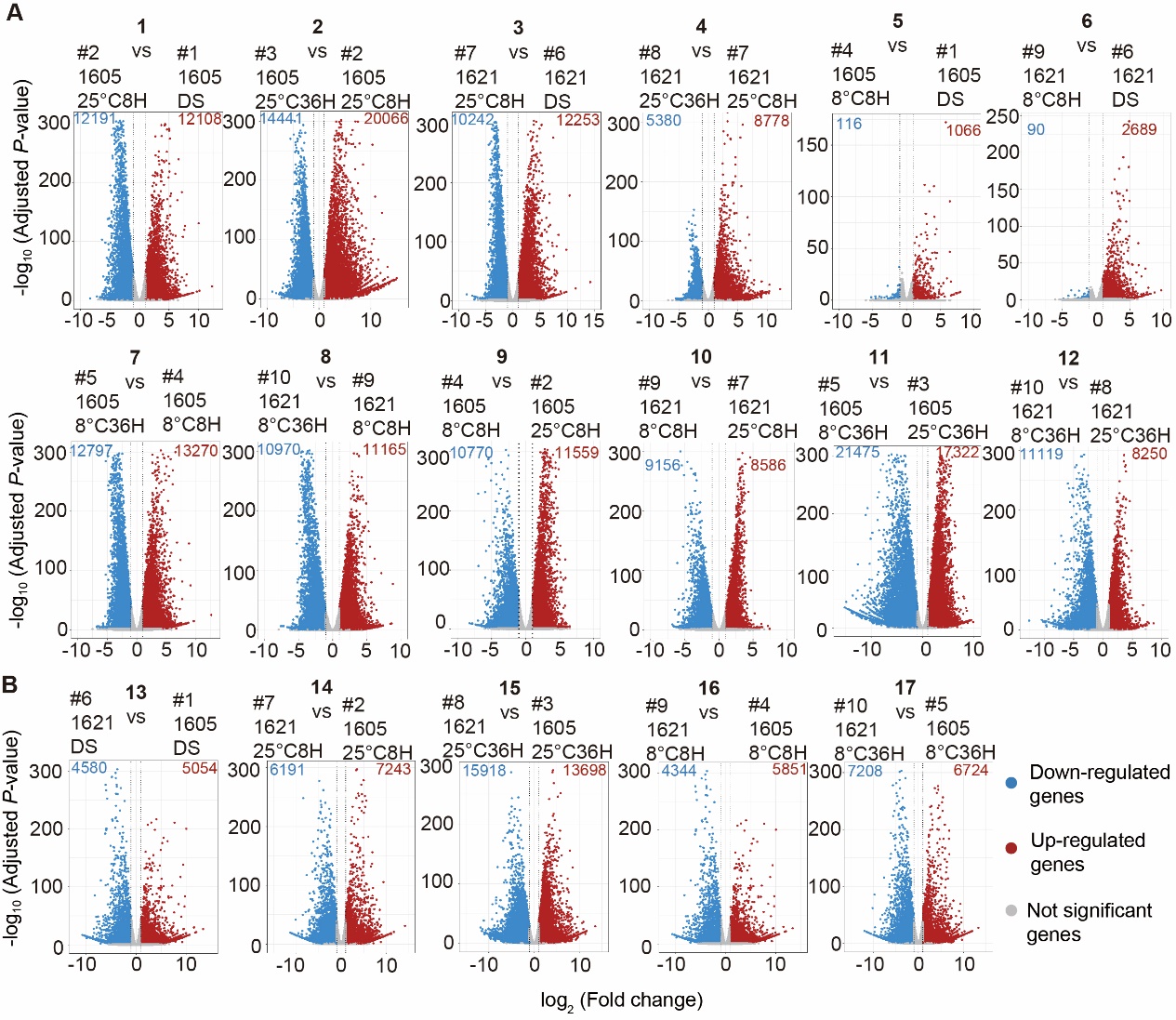


**Supplementary Figure 2. Differential gene expression analysis of normal and cold germination of JY1621 and JY1605.**

**(A)** Differentially expressed genes (DEGs) of twelve groups compared within JY1621 and JY1605. **(B)** DEGs of five groups compared between JY1621 and JY1605. 1621 is short for JY1621. 1605 is short for JY1605. Down-regulated genes, up-regulated genes and genes with no biological significance in the transcriptome analysis are labeled by blue, red and grey dots, respectively.


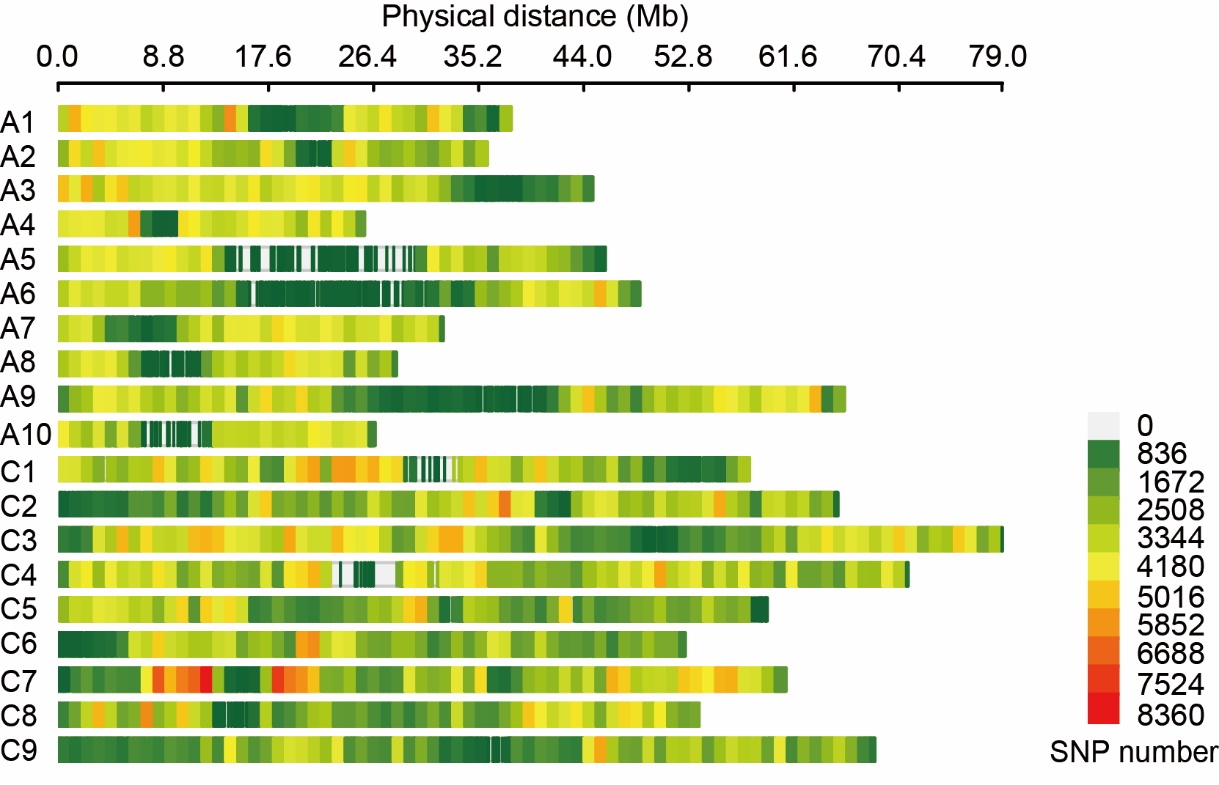


**Supplementary Figure 3. Distribution of SNPs within 1Mb window size on 19 chromosomes of 273 rapeseed accessions.**

The color bar on the right indicates the number of SNPs distributed in different regions.


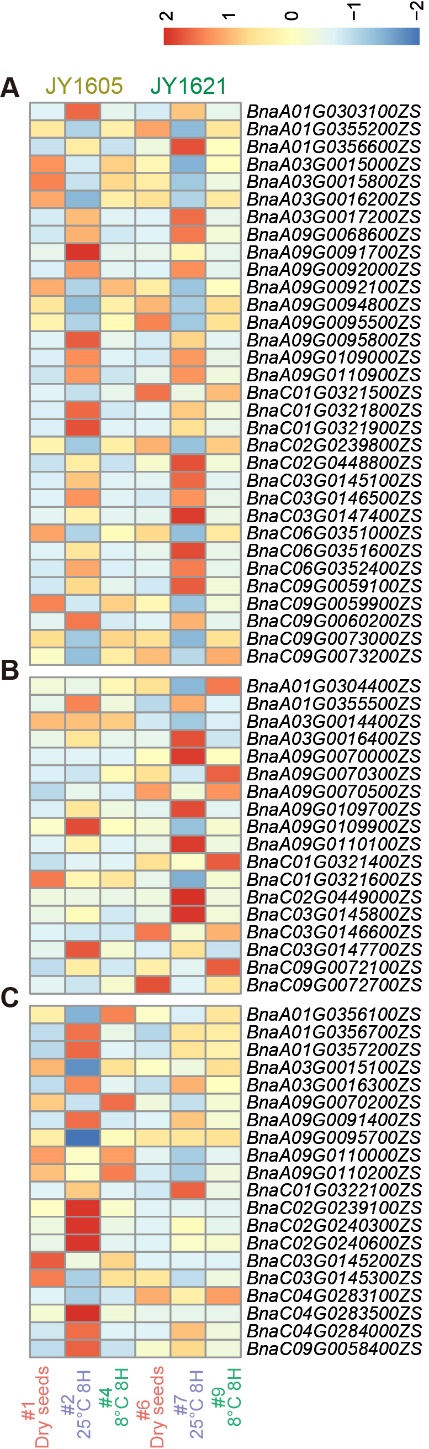


**Supplementary Figure 4.** **Heatmaps showing the expression levels of 70 genes that overlap between early NT-ETRG genes and potential germination genes from SNP-GWAS.**

**(A)** 32 genes that overlap between GWAS and NT-ETRG genes shared by both JY1621 and JY1605. **(B)** 18 genes that overlap between GWAS and NT-ETRG genes in the JY1621 dataset. **(C)** 20 genes that overlap between GWAS and NT-ETRG genes in the JY1605 dataset.


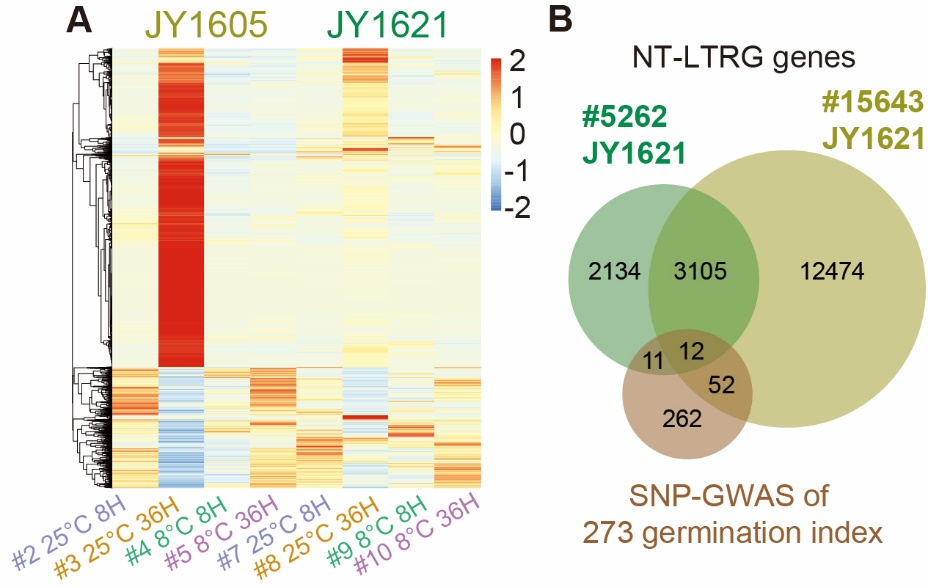


**Supplementary Figure 5. The expression profiling of NT-LTRG genes.**

**(A)** The clustered heatmap showing expression levels of totally 17788 NT-LTRG genes. **(B)** The Venn diagram represents the overlap between potential germination genes identified by SNP-GWAS and NT-LTRG genes of JY1621 and JY1605.


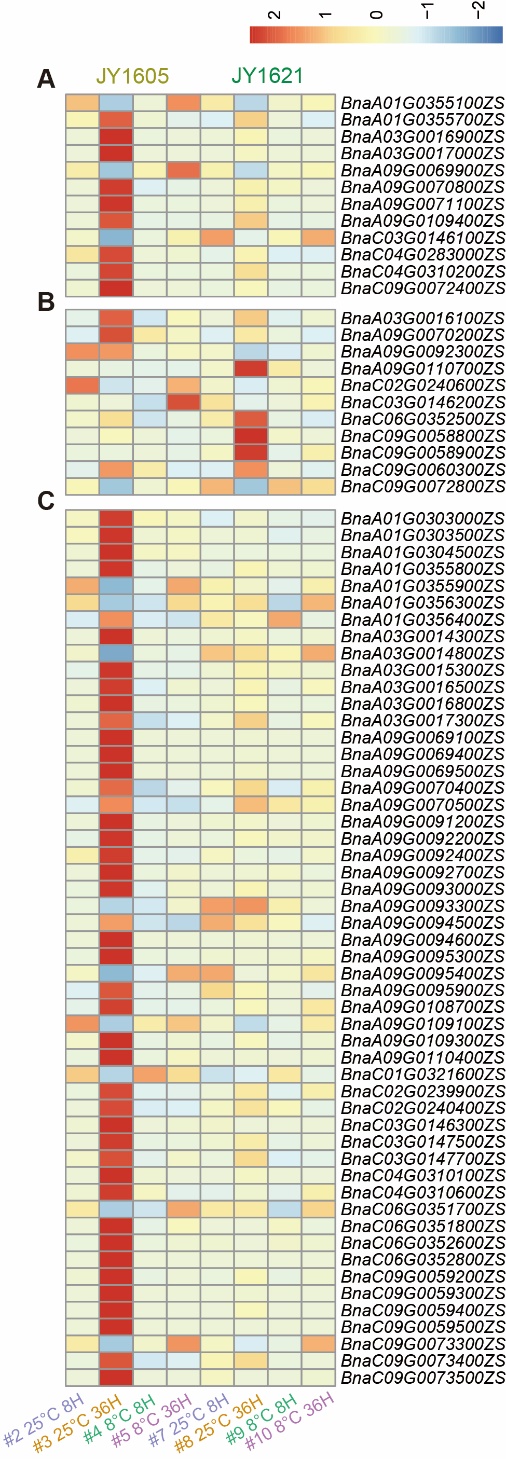


**Supplementary Figure 6. Heatmaps showing the expression levels of 75 genes that overlap between NT-LTRG genes and potential germination genes identified by SNP-GWAS.**

**(A)** 12 genes that overlap between GWAS and NT-LTRG genes shared by both JY1621 and JY1605. **(B)** 11 genes that overlap between GWAS and NT-LTRG genes in the JY1621 dataset. **(C)** 52 genes that overlap between GWAS and NT-LTRG genes in the JY1605 dataset.


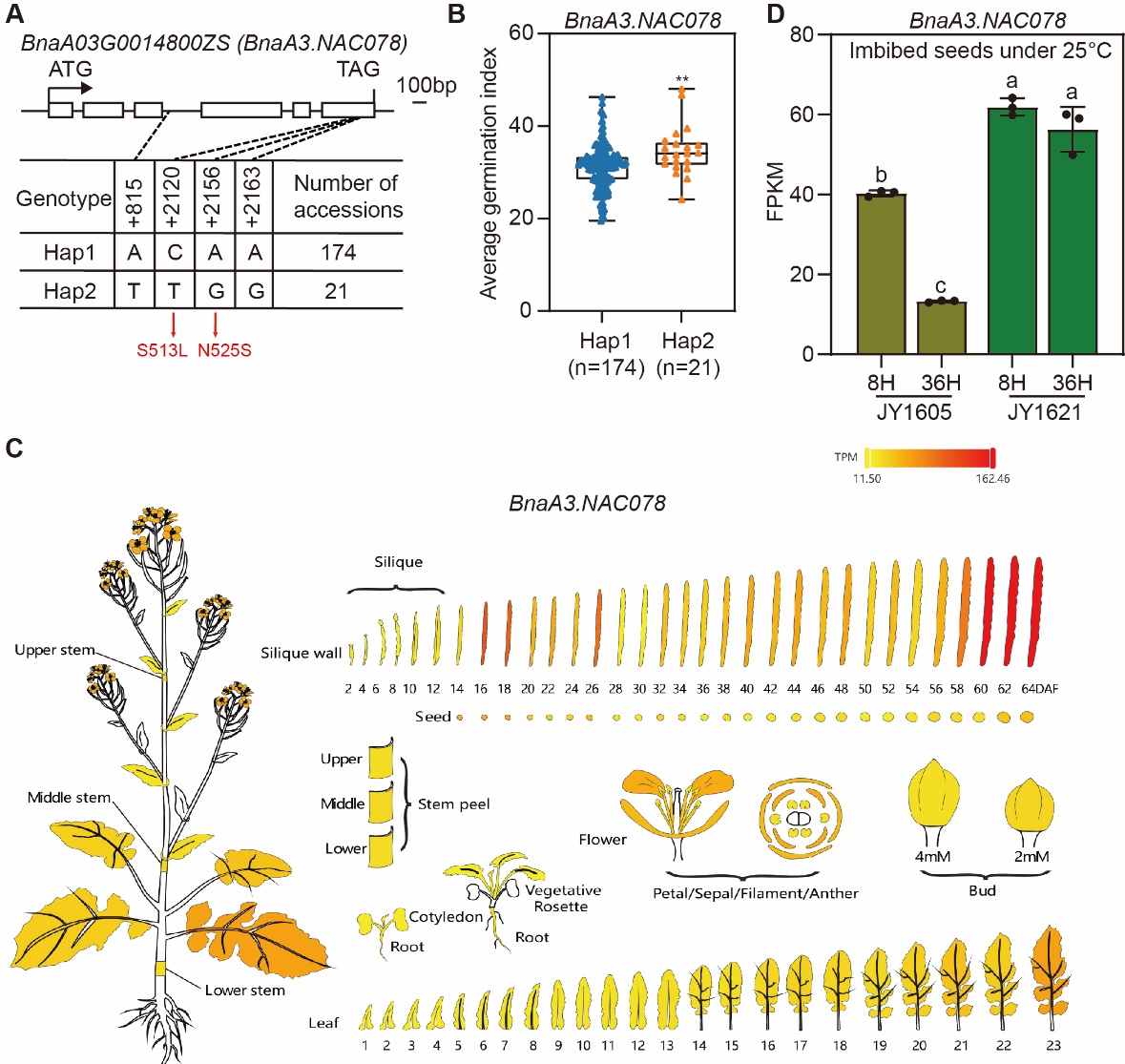


**Supplementary Figure 7. Haplotype analysis and expression patterns of *BnaA3.NAC078*.** **(A)** Diagrammatic illustration showing the gene structure (top) and two main haplotypes (bottom) of *BnaA3.NAC078* using ZS11 as the reference. S513L and N525S represent two amino acid changes caused by nonsynonymous mutations. **(B)** Comparison of average germination indices under normal-temperature conditions between accessions with two different haplotypes of *BnaA3.NAC078*. The *P*-value IS calculated by the two-tailed Student’s *t* test. ** indicates *P* < 0.01, **(C)** The tissue-specific expression pattern of *BnaA3.NAC78*. Data of expression levels in various tissues are obtained from BnIR (<https://yanglab.hzau.edu.cn/BnIR/>) (Yang et al., 2023). **(D)** Comparison of expression levels of *BnaA3.NAC078* in imbibed seeds under 25 °C between JY1605 and JY1621. Different letters represent significant differences in the one-way ANOVA.

**References in the Supplementary File**

Yang, Z., Wang, S., Wei, L., Huang, Y., Liu, D., Jia, Y., et al. (2023). BnIR: A multi-omics database with various tools for Brassica napus research and breeding. *Mol Plant* 16(4), 775-789. doi: 10.1016/j.molp.2023.03.007.
